# New Genotypes of *Coxiella burnetii* Circulating in Brazil and Argentina

**DOI:** 10.3390/pathogens9010030

**Published:** 2019-12-28

**Authors:** Mateus de Souza Ribeiro Mioni, Karim Sidi-Boumedine, Felipe Morales Dalanezi, Sâmea Fernandes Joaquim, Renan Denadai, Wanderson Sirley Reis Teixeira, Marcelo Bahia Labruna, Jane Megid

**Affiliations:** 1 Departamento de Higiene Veterinária e Saúde Pública, Universidade Estadual Paulista “Júlio de Mesquita Filho”, Botucatu, 18610-000 São Paulo, Brazil; mateus.mioni@unesp.br (M.d.S.R.M.); fm.dalanezi@unesp.br (F.M.D.); samea.joaquim@unesp.br (S.F.J.); renanvetbtu@hotmail.com (R.D.); wanderson.teixeira@unesp.br (W.S.R.T.); 2 Agence Nationale de sécurité Sanitaire de l’Alimentation, de l’Environnement et du Travail, 06902 Sophia Antipolis, France; karim.sidi-boumedine@anses.fr; 3 Instituto de Química, Universidade de São Paulo, 05508-000 São Paulo, Brazil; 4 Departamento de Medicina Veterinária Preventiva e Saúde Animal, Universidade de São Paulo, São Paulo, 05508-270 São Paulo, Brazil; labruna@usp.br

**Keywords:** *Coxiella burnetii*, Q fever, multilocus sequence typing (MST), tandem repeatsequences

## Abstract

*Coxiella burnetii*, the zoonotic agent of Q fever, has a worldwide distribution. Despite the vast information about the circulating genotypes in Europe and North America, there is a lack of data regarding *C. burnetii* strains in South America. Here, we show the presence of novel multispacer sequence typing (MST) genotypes of *C. burnetii* in two clusters detected in Brazil and Argentina that seem to be distant in parenthood. Argentinian strains isolated from a tick belongs to a new phylogenetic branch of *C. burnetii*, and the Brazilians strains may be related to MST 20 and 61. Multilocus variable number tandem repeats analysis (MLVA) typing provided a deeper resolution that may be related to host clusters of bovines, caprine, ovine, and ticks. Our results corroborate with the reports of geotypes of *C. burnetii.* Thus, we highlight the need for more genotyping studies to understand the genetic diversity of *C. burnetii* in South America and to confirm the hypothesis of host-related genotypes. We also emphasize the importance of virulence studies for a better understanding of Q fever in the region, which may help in surveillance and disease prevention programs.

## 1. Introduction

*Coxiella burnetii* is the causative agent of Q fever in humans and coxiellosis in animals [1,2], a disease that has a worldwide distribution, except for New Zealand [1,3]. In humans, Q fever is mainly asymptomatic, but acute, chronic, and more severe forms [2] are also possible outcomes of the disease. The main reservoirs of the disease for humans are domestic ruminants, where the infection is associated with late abortion and reproductive disorders. In cows, a link is suggested with metritis, placentitis, and infertility [1]. Although ticks can transmit *C. burnetii* in experimental systems, the transmission in natural environments must be rare [4].

The importance of Q fever, in terms of public health, increased after the outbreak in the Netherlands, where more than 4000 people became ill and 50,000 animals were slaughtered to control the epidemics [5,6]. Recently, we reported the presence of bacteria in Brazil by qPCR in raw cow’s milk sold directly for human consumption and its risk to human health [7]. The use of molecular typing is useful to determine the source of the outbreak, leading to more precise preventive measures [8]. Genotyping studies of *C. burnetii* isolates can enhance the ability to identify a source of infection, helping to establish preventive and control measures, reducing the number of cases in an outbreak [9], and discarding malicious use of the bacteria. Additionally, systematic typing of strains can help in the identification and follow-up of virulent lineage.

The study aimed to genotype strains of *C. burnetii* detected in Brazil and Argentina. This study is the first report of multilocus variable number tandem repeats analysis (MLVA) and multispacer sequence typing (MST) genotypes of *C. burnetii* in Brazil and Argentina.

## 2. Results

### 2.1. Multispacer Sequence Typing (MST)

**Figure 1:**
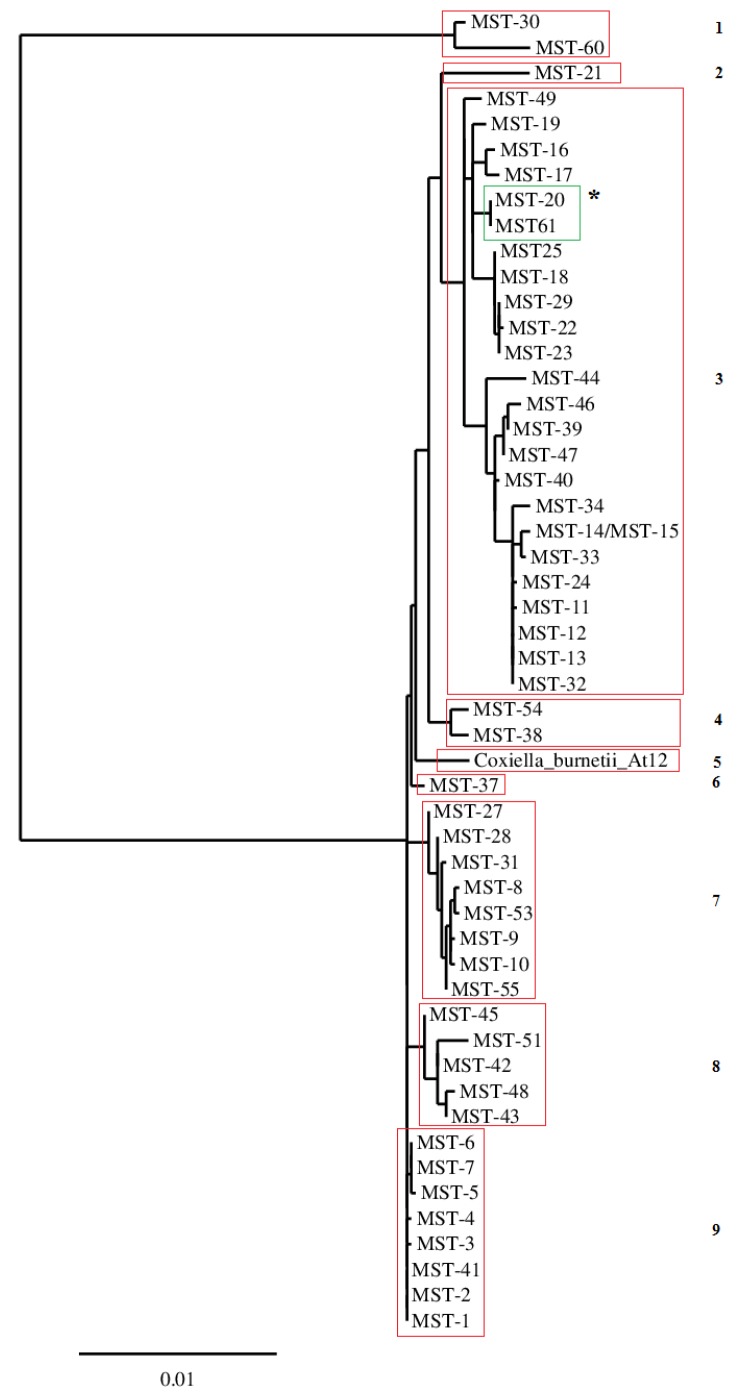
Phylogenetic tree of *Coxiella burnetii* strain *At12* based on 10 loci of MST analysis. Legend: Arabic numbers (1, 2, 3 ...) are related to the clade division. * Green color clade where Brazilian samples are placed in the phylogenetic tree using six loci.

The result of the MST is displayed in Table 1. A complete MST pattern was only possible to observe for the *C. burnetii* strain *At12*, probably due to its higher DNA concentration, once it was the only sample derived from the Vero cell culture. This strain, isolated from a tick from Argentina, is a new MST genotype and revealed different polymorphisms in Cox 20, Cox 37, Cox 51, Cox 56, and Cox57. After phylogenic analysis, the strain from Argentina was revealed to belong to a new phylogenic branch of *C. burnetii* (Figure 1).

For the three ruminant strains from Brazil, less than half of the loci were able to be sequenced with good resolution, possibly due to low DNA concentration, since it was analyzed directly from clinical samples. Table 2 summarizes the results of the quantification of the samples by qPCR. Nevertheless, with the four genotyped loci common to all ruminant strains, we believe that they are possibly the same MST clone. Moreover, Brazilian strains are also novel MST types, since its spacer sequences are diverse from the ones described among the 65 different MST types in the database (http://ifr48.timone.univ-mrs.fr/mst/coxiella_burnetii/groups.html).

**Figure 2:**
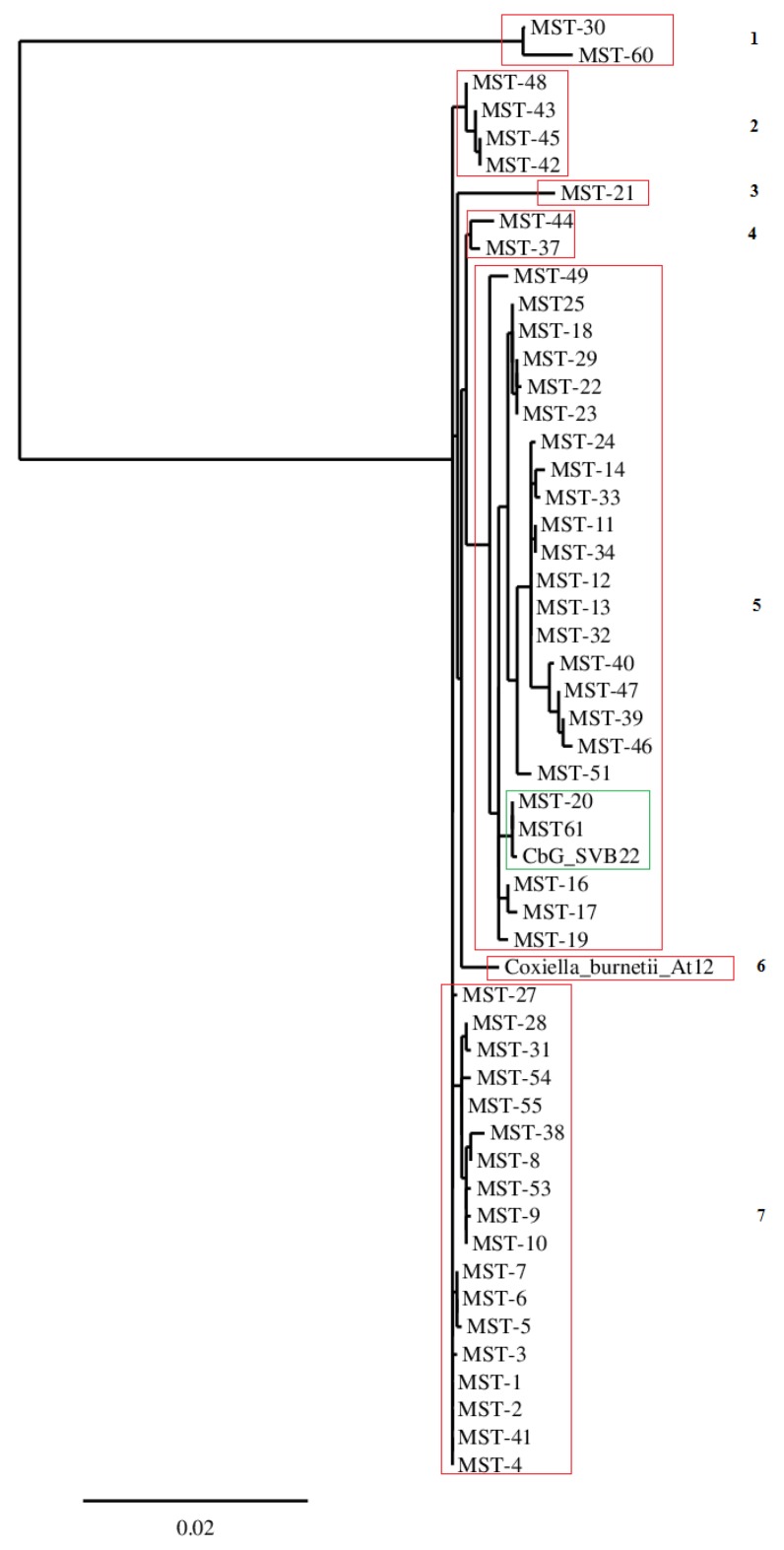
Phylogenetic tree of *Coxiella burnetii* strain *At12* and Brazilian sample CbG_SVB22 based on six loci of MST analysis. Legend: Arabic numbers (1, 2, 3 ...) are related to the clade division. Green clade is where the Brazilian samples are placed in.

A phylogenic analysis performed with the six cox spacers of sample CbG_SVB22 (Figure 2) placed the Brazilian ruminant strain close to MST 20 and MST 61, which belongs to the monophyletic group 2, according to Glazunova et al. [10]. Accordingly, in the previous phylogenetic tree constructed with all loci, the strain *At12* continued alone in a monophyletic branch of the tree drawn with only the six spacers common to CbG_SVB22 and the 65 MST types.

### 2.2. Multilocus Variable Number Tandem Repeats Analysis (MLVA)

**Figure 3:**
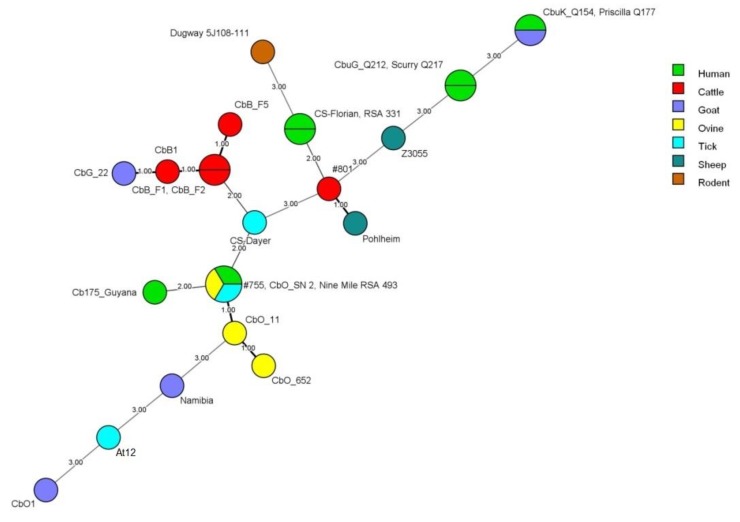
Minimum spanning tree of *Coxiella burnetii* strains based on six intergenic spacers of MLVA analysis common to all samples tested.

**Figure 4:**
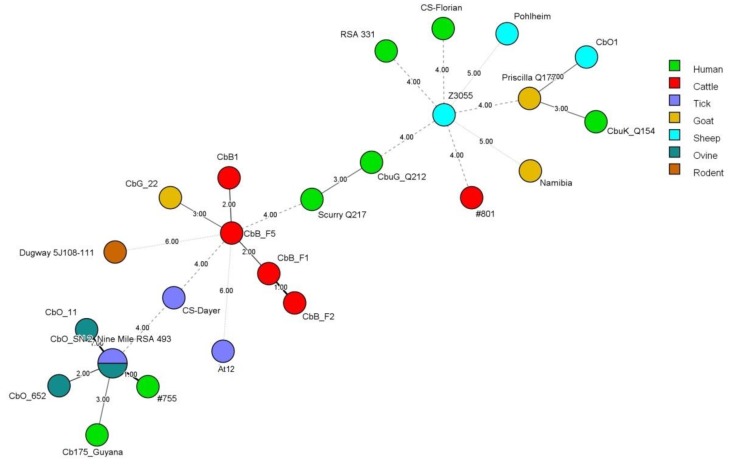
Minimum spanning tree of *Coxiella burnetii* strains using all loci of the MLVA typing analysis.

As in the MST typing, the full MLVA typing scheme was only possible for the strain *At12* (Table 3). A minimum spanning tree (Figure 3) of the MLVA analysis using the six loci common to all strains tested showed that the ruminant strains could be clustered in three different variants with separation within possible host-adapted subtypes. Strain *At12*, from Argentina, was represented far from samples from Brazil, which reinforces the separated evolution. We obtained a similar result with a minimum spanning tree built with all loci (Figure 4). All South American MLVA types were novel genotypes, once they were different from the 88 genotypes deposited in the MLVA database.

## 3. Discussion

This paper describes the genetic diversity of *C. burnetii* from Brazil and Argentina using MST and MLVA typing schemes. We demonstrate that *C. burnetii* strains circulating in some parts of those countries are novel genotypes, diverse from the genotypes already described in literature and present in public databases. Our results suggest independent evolution of the Argentine strain *At12* isolated by Pacheco et al. [11] and a common ancestor between the Brazilian strains and both MST 20 and MST 61, possibly inserted in the territory due to the trade of animals and animal products.

MST was performed for the Argentinian strain *At12* isolated from a tick [11] and for the Brazilian strains detected in fetuses from cattle (CbB_F2), and vaginal swabs were taken from a goat (CbG_SVB22) and a ewe (CbO_sn2). The combination of alleles of all strains have not been observed among 65 MST genotypes currently recognized in the Coxiella MST database, representing new genotypes of *C. burnetii* circulating in Brazil and Argentina. Furthermore, loci Cox20, Cox37, Cox51, Cox56, and Cox57 of strain *At12* revealed several polymorphisms, with Cox 56 being highly polymorphic with changes in nucleobases in positions 89, 93, 95, 132, 183, and 245. Those alleles were assigned as new Cox spacers for each locus. The polymorphism observed for the referred loci is in agreement with the observation of Glazunova et al., which reported that those spacers are highly variable [10].

The Brazilian strains from goats (CbG_SVB22), cattle (CbB_F2), and ovine (CbO_sn2) have the same MST loci combination for Cox 2, Cox 18, Cox 37, and Cox 61 and seem to be the same MST clone of *C. burnetii*. Additionally, Cox 5 is equal for CbB_F2 and CbG_SVB22, reinforcing the idea of a unique ruminant MST clone. Although some loci presented nonspecific amplification, probably due to low DNA concentration, this drawback does not have an impact on the description of new MST genotypes. This observation was sustained by MST types 35, 36, 59, and 59, which were genotypes using only the intergenic spacers Cox2, Cox5, and Cox18 (http://ifr48.timone.univ-mrs.fr/mst/coxiella_burnetii/groups.html), and also by Tilburg et al. [12]. Furthermore, the only difference between the phylogeny tree constructed with 10 loci and 6 loci is that clades four, seven, and nine of the full loci phylogenic tree were inserted in clade seven of the phylogenic tree, drawn with six spacers, indicating only a reduction in the discriminatory power.

The close relationship observed between samples from Brazil and MST 20 and MST 61 in the phylogenetic tree suggests a common ancestor, possibly due to the animal trade. The possibility of insertion of *C. burnetii* strains in Brazil through the animal trade is supported by an ancient report from 1955 of Q fever-infected bovines imported from The Netherlands and England [13]. Both, MST20 and MST61 were isolated from ruminants [14,15]. MST 20 has a widespread distribution in the United States, being the most common MST type in the country, and also has been detected across Europe and Ethiopia [16,17]. MST 61 was isolated from an abortion case in Poland [14]. In both papers, MLVA genotyping revealed further discrimination of the MST types in two or three other subtypes [14,15]. The observation of region-specific genotypes is reported in the literature [16,18,19,20], and the presence of novel genotypes in Brazil and Argentina reinforces this cartographic feature of the genetic diversity based on MST typing of Q fever.

A minimum spanning tree analysis using the MLVA typing scheme performed with 17 loci provided higher discrimination of Brazilian strains, with a separation into host-specific lineages of cattle, goats, and ovine (Figure 4). The observation of host-adapted strains using MLVA analysis has been described in France by Joulié et al. [21], and also by Tilburg et al. [12], who described cattle-adapted strains in Europe. In the minimum spanning tree, Brazilian strains derived from cattle and goats showed a close pattern with the French strain, CbB1, detected in cattle placenta [9]. The Argentinian strain was placed far from the ruminant strains from Brazil. Some loci from Brazilian samples presented flaws of amplification, probably due to the low concentration of the samples, as observed in Table 2.

Because the lack of data for some loci can lead to impairment of kinship between strains, a minimum spanning tree was drawn with the six genotyped loci common to all strains in the MLVA analysis (ms21, ms22, ms23, ms28, ms33, and ms34) (Figure 3), decreasing the discriminatory power. This approach allowed the observation of possible host-adapted lineage and the proximity between cattle and goat strains from Brazil and its similarity with the French strain, CbB1, while the ovine strains are linked in proximity to Cb175_Guiana (MST 17), Nine Mile (MST 16), and #755 (MST 16) from French Guiana, USA, and Poland, respectively. Comparing the six loci (ms21, ms22, ms23, ms28, ms33, and ms34) in the MLVA database resulted in novel MLVA genotypes. Cattle and goat samples from Brazil presented fingerprints more related to MLVA6 Nijmegen type 10 (CbB1 and Cbg1506), which were isolated from cattle and goats in France [9], while Brazilian ovine samples were closer to MLVA6Nijmegen types 1 (Nine Mile) and 33 (Cb175_Guyana), isolated from ticks and humans [9,20]. Strain *At12* had a fingerprint that resembles types 66 (CbO1) and 75 (Namibia) of the Nijmegen scheme.

The use of molecular techniques for the discrimination of strains can help in the understanding of the epidemiology of diseases and is a suitable tool for outbreak traceability. The presence of novel MST and MLVA genotypes of *C. burnetii* in Brazil and Argentina raises the question of the importance of different lineages of *C. burnetii* in animal and human cases of Q fever, with a necessity of virulence studies to elucidate this question. The report of abortion in goats due to *C. burnetii* [22] and Q fever in humans in Brazil and Argentina [23,24,25,26] indicates the presence of virulent strains circulating in those countries.

This is the first genotype study of *Coxiella* strains from Brazil and Argentina using MST and MLVA, molecular tools commonly used for this pathogen. Those techniques allow the comparison between strains and the traceability of the source of outbreaks of Q fever [8,27], helping to develop control and preventive measures for the disease and a better understanding of the epidemiology of the disease. Since cases of Q fever have been reported in Brazil and other countries of South America, more genotype, isolation, and virulence studies of strains are necessary to better understand the situation of Q fever in this region and the diversity of the pathogen.

## 4. Materials and Methods

### 4.1. Ethics Disclosure

The research was approved by the Ethics Committee on Animal Use (CEUA-FMVZ-UNESP/Botucatu, State of Sao Paulo), Brazil, protocol number 0203/2016.

### 4.2. Samples

We used seven samples from Brazil, detected in three aborted fetuses (pool of organs) from dairy cows, three vaginal swabs from ewes, one vaginal swab from a goat, and one tick isolate, strain *At12* [11] from Argentina. All vaginal swabs from Brazil were sampled in flocks of the São Paulo State University (UNESP) with a report of abortion. Samples from abortions were sent to the laboratory of infectious diseases diagnosis of UNESP from 2013 to 2017. The clinical samples were selected through quantitative PCR (qPCR) for *C. burnetii*, and only samples with more than 10^3^ bacteria per milliliter were used for MLVA typing. Because MST is based on sequencing analysis, only samples with more than 10^4^ were typed through this method.

### 4.3. Quantitative PCR (qPCR)

We performed qPCR, targeting the *IS1111* element with specific primers and probes developed by ANSES (Sophia Antipolis, France) and produced by GENEWIZ (Leipzig, Germany). The quantitative PCR reaction was performed in a total volume of 25 µL, as described by Mioni et al. [7]. A standard curve ranging from 2 × 10^2^ to 2 × 10^7^ genome equivalent (GE) bacteria/mL was used for quantification proposes.

### 4.4. Multispacer Sequence Typing (MST)

MST typing was performed using primers described by Glazunova et al. [10] for 10 loci markers amplified via polymerase chain reaction (PCR). PCR amplification was performed as follows: 33.5 µL of water, 1X of Phusion HF BufferTM, 200µM of dNTP, 0.5µM of each forward and reverse primer, 0.02 U/µL of Phusion DNA polymerase, and 2µL of a DNA template. Cycling conditions were composed by 98 ºC/30 s, 40 cycles of 98 ºC/10 s, 57 ºC/30 s, and 72 ºC/1 min and an extension step of 72 ºC/10 min and held at 4 ºC. PCR products were sent to Sanger Sequencing GENEWIZ (Leipzig, Germany), and results were then analyzed using Bioedit^TM^ (Ibis Bioscience, Carlsbad, CA, USA) to create a consensus sequence based on each forward and reverse primer. Consensus sequences of each Cox spacer were then blasted and compared on the website (http://ifr48.timone.univ-mrs.fr/mst/coxiella_burnetii/) of the institution Mediterranée Infection. The combination of all loci was then used to assign the MST type.

### 4.5. Sequencing Analysis

All DNA sequences generated during the experiment were deposited at GenBank. This included strain *At12* (accession number MK502121–MK502130), CbB_F2 (accession number MK502135–MK502139), CbG_SVB22 (accession number MK168659–MK168664), and CbO_sn2 (accession number MK502131–MK502134).

### 4.6. Multilocus Variable Tandem Number Repeats Analysis (MLVA)

For the MLVA, we used 17 markers and coding, according to the protocol of Arricau-Bouvery et al. [9], complemented by the new MLVA recommendations defined by Université de Paris-Sud (http://mlva.u-psud.fr/MLVAnet/spip.php?rubrique50). PCR was used for all variable number of tandem repeat (VNTR) loci amplification, based on the procedure described by Arricau-Bouvery [9] and Prigent et al. [28], with primers described on the abovementioned website. PCR products were analyzed through the Agilent 2100 Bioanalyzer (Agilent Technologies, Santa Clara, USA) using a DNA 7500 LabChip^®^kit, following the manufacturer’s instructions. A *C. burnetii* nine mile strain was used as the reference in all the experiments for normalization and for comparing the MLVA profiles obtained. According to Svraka et al. [28], and as recommended by the Université de Paris-Sud (http://mlva.u-psud.fr/MLVAnet/spip.php?rubrique50), for each marker, the fragment length of the sample was compared to the fragment length of the reference strain for determining the number of repeats. According to the same authors and the website, the genotypes were considered distinct from each other when the number of repeats of at least one of the markers differed. The MLVA pattern of the isolates was compared in the database (http://mlva.u-psud.fr/mlvav4/genotyping/) to check similarities with other isolates.

### 4.7. Minimum Spanning Tree Analysis

The software BioNumerics version 7.5 (Applied Maths NV, Sint-Martens-Latem, Bélgica) (Available from http://www.applied-maths.com) was used to create a minimum spanning tree with the following strains from goats, ewes, cattle, ticks, and humans: Namibia, Priscilla Q177, Z3055, #801, CbuG_Q212, Cb175, RSA331, #755, CS-Florian, CbuK_Q154, Scurry Q217, CS-Dayer, Nine Mile, and Dugway 5J108-111. All MLVA patterns for these strains can be found at the public database MLVA bank (http://microbesgenotyping.i2bc.paris-saclay.fr/databases/view/966).

### 4.8. Phylogeny Tree Construction

Phylogenetic relationships were assessed using the website (phylogeny.fr) [29]. Briefly, a multiple sequence comparison by log-expectation (MUSCLE) alignment of the sequences (version 3.8.31_2) [29,30], followed by G blocks curation (version 0.91.1) [29,31] for cleaning aligned sequences was performed. Subsequentially, to construct the phylogenic tree, phylogeny and the tree rendering were performed using phylogeny software based on the maximum-likelihood (PhyML, version 3.1_1) [29,32], dynamic graphics, and annotations for phylogenetic analyses of trees (TreeDyn, version 198.3) [29,33,34]. The robustness of the generated tree was evaluated through a comparison with the phylogenic tree available at the MST database for *C. burnetii*.

## Figures and Tables

**Table 1:** Results of *Coxiella burnetii* genotyping based on multispacer sequence typing (MST).

			MST Loci									
Strain	Host	Country	Cox 2	Cox 5	Cox 18	Cox 20	Cox 22	Cox 37	Cox 51	Cox 56	Cox 57	Cox 61
Cb_*At12*	Tick	Argentina	3	6	8	New	6	New	New	New	New	6
CbO_sn2	Sheep	Brazil	3	PSA	5	PSA	PSA	10	PSA	PSA	PSA	5
CbB_F2	Cattle	Brazil	3	2	5	PSA	PSA	10	PSA	PSA	PSA	5
CbG_SVB22	Goat	Brazil	3	2	5	PSA	PSA	10	PSA	10	PSA	5

Legend: New, locus did not match with previous described MST due to polymorphism in the nucleobases; poor sequence assembly (PSA).

**Table 2:** Summary of qPCR of the samples used in multilocus variable number tandem repeats analysis (MLVA) and MST typing.

Identification	Host	Source	Ct in qPCR	Quantification in qPCR (*C. burnetii*/mL)
*At12*	Tick	Cell culture	19.72	>10^7^
CbB_F1	Bovine	Fetus	36.11	5.69 × 10^2^
CbB_F2	Bovine	Fetus	33.24	9.58 × 10^3^
CbB_F5	Bovine	Fetus	31.00	1.57 × 10^4^
CbG_SVB22	Caprine	Vaginal swab	31.9	2.96 × 10^3^
CbO_11	Ovine	Vaginal swab	32.24	4.89 × 10^3^
CbO_sn2	Ovine	Vaginal swab	23.34	2.42 × 10^6^
CbO_652	Ovine	Vaginal swab	29.52	3.26 × 10^4^

**Table 3:** Results of *Coxiella burnetii* analysis based on the multilocus variable tandem repeat analysis (MLVA).

			MLVA Loci																
Strain	Host	Country	ms01	ms03	ms07	ms12	ms20	ms21	ms22	ms23	ms24	ms26	ms27	ms28	ms30	ms31	ms33	ms34	ms36
At12	Tick	Argentina	4	6	6	8	18	7	6	3	7	2	2	5	12	3	9	2	11
CbB_F1	Cattle	Brazil	F/A	F/A	F/A	6	15	6	6	6	18	F/A	2	7	12	3	9	9	4
CbB_F2	Cattle	Brazil	3	6	8	6	15	6	6	6	18	F/A	3	7	12	3	9	9	4
CbB_F5	Cattle	Brazil	3	6	F/A	F/A	15	6	6	6	12	F/A	2	7	12	F/A	9	10	4
CbO_11	Ovine	Brazil	F/A	7	11	7	13	9	6	9	27	8	F/A	6	12	F/A	9	5	F/A
CbG_SVB22	Goat	Brazil	F/A	6	8	7	15	6	6	6	NA	8	2	7	19	3	9	11	4
CbO_sn2	Ovine	Brazil	4	7	F/A	F/A	F/A	6	6	9	27	F/A	4	6	12	5	9	5	F/A
CbO_652	Ovine	Brazil	F/A	7	F/A	10	16	9	6	9	27	F/A	4	6	F/A	5	6	5	F/A
Nine Mile	Tick	USA	4	7	8	8	15	6	6	9	27	4	4	6	12	5	9	5	4

Legend: Fail in amplification (F/A), probably due to low *C. burnetii* DNA concentration.
